# A survey of physicians and physiotherapists on physical activity promotion in Nigeria

**DOI:** 10.1186/s40945-017-0034-8

**Published:** 2017-05-19

**Authors:** Adewale L. Oyeyemi, Adetoyeje Y. Oyeyemi, Rahana Y. Habib, Rashida B. Usman, Jasper U. Sunday, Zubair Usman

**Affiliations:** 1 Department of Physiotherapy, College of Medical Sciences, University of Maiduguri, P.M.B 1069 Bama Road, Maiduguri, Borno State Nigeria; 2 Faculty of Medicine, Federal University Dutse, Dutse, Jigawa State Nigeria

**Keywords:** Physical inactivity, Exercise, Health promotion, Health professionals, Non-communicable diseases

## Abstract

**Background:**

Effective control of non-communicable diseases and promotion of population-wide physical activity participation require the active engagement of health professionals. Physiotherapists and physicians, as part of their practice, routinely screen and assess physical activity status, and recommend health enhancing physical activity participation for their patients. This study aims to compare Nigerian physiotherapists and physicians’ knowledge of physical activity message, role perception and confidence, perceived feasibility and barriers, and overall disposition to promoting physical activity in their practice.

**Methods:**

A total of 153 physicians and 94 physiotherapists recruited from 10 government hospitals in five states in Northern Nigeria completed a standardized physical activity promotion questionnaire that elicited information on the knowledge of physical activity, role perception and confidence, feasibility, and barriers to physical activity promotion. Descriptive and inferential statistics were used to analyze the data.

**Results:**

The physiotherapists and physicians were fairly knowledgeable on physical activity message (14.2 ± 2.1/20), reported minimal or little barrier to physical activity promotion (23.7 ± 3.1/30), perceived physical activity promotion as their role (13.0 ± 1.8/15), were confident in their ability to discuss and recommend physical activity promotion (7.6 ± 1.6/10) and believed promoting physical activity was feasible for them (15.6 ± 2.6/20). However, over 40% of the physiotherapists and physicians do not know the correct dosage of physical activity that could confer health benefits to patients. The physicians showed better overall disposition to physical activity promotion than the physiotherapists (*P* = 0.048), but more physiotherapists than the physicians believed ‘*it is part of their role to suggest to patients to increase their daily physical activity’* (95.7% vs 88.2%, *P* = 0.043) and were more *‘confident in suggesting specific physical activity programs for their patients’* (87.2% vs 64.5%, *P* < 0.001).

**Conclusion:**

Physiotherapists and physicians in Nigeria demonstrated good disposition to promoting physical activity but many of them have knowledge deficits on the correct dosage required for better health for their patients. These health professionals can serve as good advocates for physical activity promotion in Nigeria, but many of them may require knowledge update on health enhancing physical activity for effective health promotion and primary prevention of non-communicable diseases.

**Electronic supplementary material:**

The online version of this article (doi:10.1186/s40945-017-0034-8) contains supplementary material, which is available to authorized users.

## Background

Physically active lifestyles have been shown to significantly reduce the risk of developing cardiovascular diseases, obesity, type 2 diabetes, several forms of cancer, dementia and premature deaths [1–3]. It has been established that participation in at least 150 min of moderate-intensity or 75 min of vigorous-intensity physical activity per week, or an equivalent combination, decreases all-cause mortality risk by 20–30% compared to insufficient physical activity [4, 5]. Also, there is evidence that participation in moderate-to vigorous physical activity at leisure time can provide important health benefits compared to a sedentary lifestyle [6, 7]. Due to the rapidly rising prevalence of chronic non-communicable diseases in developing countries [1, 8], it is imperative to prioritize physical activity promotion as an important public health agenda in African countries [9, 10].

Despite the overwhelming evidence on the benefits of physical activity in the prevention, treatment, and rehabilitation of major public health diseases [11–14], physical activity levels remain low worldwide [3, 15] and vary across populations [16]. Thus, there is a compelling need to promote physical activity participation in the global population [3]. In this context, policy interventions that can bring about population wide change in physical activity participation have been instituted in many countries [3]. However to assist with policy and programme implementation, health professionals with requisite knowledge and expertise are required to participate in effecting positive changes in physical activity behavior in many developing countries [17, 18].

Traditionally, nurses, physiotherapists and physicians are among the healthcare professionals involved in the primary prevention of non-communicable diseases or risk reduction for these diseases. Due to their presumed expertise on health promotion, physiotherapists and physicians are however more likely to be asked for advice or consulted on physical activity than other healthcare professionals [19–22]. In Australia, for example, it was recognized that in getting Australians to be active, physicians and other health care providers have to be involved in physical activity promotion [23]. Hence, interest on the health professionals’ awareness of physical activity guidelines and their disposition to promoting health enhancing behaviors among their clients and patients has been on the rise [24, 25].

Health professionals’ disposition to promoting physical activity is an indirect indication of their knowledge and understanding of the health enhancing benefits of physical activity and their role in its promotion. Some studies show that physicians do not discuss physical activity with majority of their patients [19, 26]. However, physicians in Australia had good knowledge of the benefits of regular physical activity and the required level for good health [20]. Canadians physicians reported they routinely ask their patients about physical activity and provide counselling [27], but they also identified lack of time as a possible barrier to promoting physical activity among their patients [19, 27]. Similarly, the roles of physiotherapists in physical activity promotion have been documented in studies among physiotherapists in Australia [21], Germany [22], United Kingdom [28] and the United States [29]. Given their commitment to exploiting effective noninvasive interventions, physiotherapists are considered to be in a preeminent position to promote physical activity in their practice setting and among the general population [30, 31].

It can be argued that the disposition of physiotherapists and physicians to recommend physical activity is an indication of their awareness of the menace of non-communicable diseases and solution to mitigating these problems. Because any advice by physiotherapists and physicians on health promotion would likely be followed by patients and clients [24, 25, 29–31], understanding the disposition of these health professionals to recommending health enhancing physical activity is important to formulating strategies to improve physical activity behavior in the population. Since majority of the available studies on this topic were conducted mainly in Western high income countries, it is unclear whether the findings from these studies can be generalized to low- and middle income- countries. Presently, no published study has compared the disposition of physiotherapists and physicians to physical activity promotion, and there is dearth of empirical data on the knowledge and disposition of Nigerian physiotherapists and physicians to physical activity and health promotion recommendations. In Nigeria, about 22% of the adults’ population do not participate in regular physical activity to the recommended levels (at least 150 min of moderate-intensity or 75 min of vigorous-intensity per week) and no physical activity surveillance system or any national plan and policy on physical activity exists for the country [32]. Therefore, understanding strategies to promote and improve physical activity participation in the Nigerian population is an important priority for the country. This report compared Nigerian physiotherapists and physicians’ knowledge, confidence, role perception, feasibility and barriers to physical activity promotion in their practice, and their general disposition to promoting physical activity among their patients using a combined data from two separate surveys of physicians and physiotherapists.

## Methods

### Participants, design and setting

A total of 153 physicians from two government hospitals and 94 physiotherapists from eight government hospitals in Nigeria were recruited as a convenience sample to participate in the study. The hospitals were selected across five states in Northern Nigeria and include University of Maiduguri Teaching Hospital and Maiduguri Specialist Hospital in Borno State; Federal Medical Center Yola in Adamawa State; Murtala Mohammed Specialist Hospital Kano, Muhammad Abdullahi Wase General Hospital Kano, Aminu Kano Teaching Hospital, and National Orthopedic Hospital Dala in Kano State; Federal Medical Centre Gombe in Gombe State, and Federal Medical Centre Birnin Kudu in Jigawa State. The response rate for the physicians was 84.5% (153 out of 181 contacted to participate) and 100% for the physiotherapists.

### Measures and procedure

The instrument used for data collection was adapted from a previously validated questionnaire used in a study of primary care physicians in Australia [20]. The adaptations made to the questionnaire were minor and only aimed to make the questionnaire also applicable to the physiotherapists. To ascertain the reliability of the adapted instrument prior to the main study, its two-week test-retest reliability was evaluated in a subsample of the participants (22 physicians and 15 physiotherapists). The reliability coefficients (Spearman rho) of the adapted instrument were very good (>0.90) among Nigerian physicians and physiotherapists.

The adapted questionnaire includes five subscales that assess knowledge of physical activity messages (Knowledge, 4- items), perception of their role to promote physical activity (role perception, 3-items), confidence in giving advice on physical activity (confidence, 2-items), barriers to physical activity promotion (barriers, 6-items) and feasibility of physical activity promotion strategies (feasibility, 4-items). The survey also included a single question on ‘optimal physical activity recommendations’ for adults with only one correct score out of four available response options (See Additional file 1). Possible responses to items on all the subscales ranged from a minimum score of 1 (strongly disagree or never or totally unfeasible) to 5 (strongly agree or very often or highly feasible). Generally, all the five subscales were computed as the mean of responses to items in the subscale, with responses coded (or reverse-coded) such that higher values indicated better or positive scores.

To create an overall physical activity promotion disposition (total composite scale) score, we summed the participants’ responses (scores) on the five subscales and the single question on ‘optimal physical activity recommendations’. The overall disposition score ranges from a minimum score of 20 to a maximum possible score of 100. The higher the score, the better the disposition to physical activity promotion among the physicians and physiotherapists. The term ‘disposition’ was operationally defined as the degree of willingness or agreement to promoting physical activity. We hypothesized that physicians and physiotherapists with better scores on knowledge, role perception, confidence, feasibility and perceived barriers to physical activity promotion will be more positively disposed or willing to promote physical activity in their practice.

Self-reported socio-demographic information such as age, gender, and designated professional rank was also collected from the participants. The questionnaire was self-administered and delivered in person by two of the investigators (RBU and RYH) or through contacts who were physiotherapists in the selected hospitals. It took about 10 to 15 min to complete the questionnaire. All participants provided written informed consents before participating in the study. The completed questionnaires were returned in sealed envelopes directly to two of the investigators (RBU and RYH) or through the contacts who either mailed them back to researchers’ address or picked up at a suitable time not more than four weeks following distribution of the questionnaires. The study was approved by the Research and Ethics Committee of the University of Maiduguri Teaching Hospital, Maiduguri, Nigeria.

### Statistical analyses

Descriptive statistics of mean, standard deviation and frequency were used to summarize the socio-demographic information. Independent sample t-test was utilized to determine differences in summary mean scores of the knowledge, confidence, role perception, barriers and feasibility of physical activity promotion subscales, and the overall physical activity disposition score between the physiotherapists and physicians. Health professionals’ group differences in individual items within subscales were explored with Chi-square statistics. Differences in the subscales and composite physical activity promotion scores by gender, and years of working experience within the groups was also explored at an alpha level set at *p* < 0.05.

## Results

### Socio-demographic characteristics of the participants

Participants were 247 Nigerian health professionals comprising 94 physiotherapists (39.1%) and 153 physicians (61.9%). More male (*n* = 169, 68.4%) than female (*n* = 78, 31.6%) health professionals participated in the study. Thirty-three (35.1%) of the 94 physiotherapists were females, while 45 (29.4%) of the 153 physicians were females. Ninety-eight of the participants (39.7%) were aged 30 years or below, while 149 (60.3%) were above 30 years old. While majority of the physicians were older than 30 years (77.1%), most of the physiotherapists were 30 years or younger (64.9%) (Table 1).

**Table 1:** Socio-demographic characteristics of the participants

Variable	Physiotherapist (*n* = 94)	Physician (*n* = 153)	Combined
	n (%)	n (%)	n (%)
Gender			
Male	61 (64.9)	108 (70.6)	169 (68.4)
Female	33 (35.1)	45 (29.4)	78 (31.6)
Age group			
≤ 30	61 (64.9)	35 (22.9)	96 (38.9)
≤ 31	33 (34.2)	118 (77.1)	151 (61.1)

### Knowledge, confidence and role perception on physical activity promotion message

Comparisons of the summary scores on knowledge, confidence and role perception to promoting physical activity between the physicians and physiotherapists are shown in Table 2. The summary mean scores on knowledge were 13.1 ± 3.5 and 14.7 ± 2.2 for the physiotherapists and the physicians respectively, out of the possible score of 20. Out of the possible score of 10, the summary mean score on confidence was 7.1 ± 1.8 for the physiotherapists and was 7.9 ± 1.3 for the physicians. The summary mean scores on role perception for the physiotherapists and physicians were 13.2 ± 1.9 and 12.9 ± 1.6 respectively, out of the possible score of 15. There was no significant difference (*P* > 0.05) in the knowledge, confidence and role perception summary scores between the two groups.

**Table 2:** Physical activity promotion scores in overall sample and comparison between physicians and physiotherapists

Variable	Combined (*n* = 247)	Physicians (*n* = 153)	Physiotherapists (*n* = 94)	*p*-value
Knowledge	14.2 ± 2.1/20	14.7 ± 2.2/20	13.1 ± 2.2/20	0.427
Confidence	7.6 ± 1.6/10	7.9 ± 1.3/10	7.1 ± 1.8/10	0.773
Role perception	13.0 ± 1.8/15	12.9 ± 1.6/15	13.2 ± 1.9/15	0.641
Barrier	23.7 ± 3.1/30	24.4 ± 2.5/30	23.2 ± 3.6/30	0.562
Feasibility	15.6 ± 2.6/20	15.0 ± 2.5/20	15.8 ± 2.8/20	0.733
Overall disposition	75.4 ± 7.3/100	78.5 ± 6.7/100	72.5 ± 7.9/100	0.048*

Knowledge denotes knowledge of physical activity message; Role perception denotes role perception on physical activity promotion; Confidence denotes confidence in giving physical activity message; Barriers denotes perceived barriers to physical activity promotion, Feasibility denotes feasibility of physical activity promotion strategies and Disposition denotes overall disposition to promoting physical activity

* = Significant difference at *p* < 0.05

The physicians compared to the physiotherapists tend to score better on the knowledge item *‘Physical activity that is good for health must make you puff and pant’* (16.3% 28.7% vs, *P* = 0.020) and the role perception item *‘Discussing the benefit of a physically active lifestyle is part of my role’* (97.4% vs 91.5%, *P* = 0.036). However, the physiotherapists tend to score better than the physicians on the role perception item *‘Suggesting to patients ways to increase daily physical activity is part of my role’* (95.7% vs 88.2%, *P* = 0.044) and on the confidence item *‘I would feel confident in suggesting specific physical activity programs for my patients’* (87.2% vs 64.7%, *P* < 0.001) (Table 3).

**Table 3:** Comparison of physical activity promotion subscales items scores between physicians and physiotherapists

Variable	Physicians (*n* = 153), n Agree (%)	Physiotherapists (*n* = 94), n Agree (%)	Chi-Square	*P*-value
Knowledge of PA message				
Taking the stair at work and generally being more active each day is enough physical activity to improve health	37 (24.3)	16 (17.0)	1.772	0.183
Half an hour of working on most days is all the physical activity that is needed for good health	53 (34.6)	25 (26.9)	1.744	0.187
Physical activity that is good for health must make you puff and pant	25 (16.3)	27 (28.7)	5.372	0.020*
Several short walks of 10 min each on most days is better than one session of golf or soccer per week for good health	108 (70.6)	65 (69.1)	0.057	0.811
Role perception in PA promotion				
Discussing the benefits of a physically active lifestyle with patients is part of my role	149 (97.4)	86 (91.5)	4.379	0.036*
Suggesting to patients ways to increase daily physical activity is part of my role	135 (88.2)	90 (95.7)	4.047	0.044*
I should be physically active to act as a role model for my patients	138 (90.2)	89 (94.7)	1.574	0.210
Confidence in promoting PA				
I feel confident in in giving general advice to patients on physically active lifestyle	140 (91.5)	87 (92.6)	0.086	0.769
If feel confident in suggesting specific physical activity programs for my patients	99 (64.7)	82 (87.2)	15.092	<0.001**
Barriers to PA promotion				
Lack of time	129 (84.3)	58 (61.7)	16.186	<0.001**
Lack of counselling skills	31 (20.3)	27 (28.7)	2.230	0.128
Lack of remuneration for promoting physical activity	20 (13.1)	20 (21.3)	2.888	0.089
Lack of interest in promoting physical activity	43 (28.3)	32 (34.0)	0.907	0.341
Feeling it would not change the patient’s behavior	27 (17.6)	24 (25.5)	2.209	0.137
Feeling it would not be beneficial for the patient	16 (10.5)	18 (19.1)	3.705	0.054
Feasibility of PA promotion strategies				
Brief counselling integrated into your regular consultation	141 (92.2)	82 (87.2)	1.609	0.205
Separate one-on-one consultations	84 (54.9)	66 (70.2)	5.723	0.017*
Group session	88 (57.5)	71 (75.5)	8.240	0.004**
Distribution of resources (e.g., brochures)	101 (66.0)	57 (60.6)	0.730	0.393

*PA* Physical Activity; * = *P* < 0.05; ** = *P* < 0.01

### Physical activity promotion barriers and role feasibility

Table 2 shows the comparisons of summary scores of barriers to physical activity promotion and role feasibility between the physicians and the physiotherapists. The summary mean score on barriers for the physiotherapists was 23.2 ± 3.6 out of a possible score of 30, while it was 24.4 ± 3.5 for the physicians. The summary mean feasibility score was 15.8 ± 2.8 out of a possible score of 20 for the physiotherapists, while the score for the physicians was 15.0 ± 2.5. No significant difference (*P* > 0.05) was observed for the summary scores on barriers and role feasibility between the physicians and the physiotherapists. *‘Lack of time’* was more frequently cited as a barrier to physical activity promotion by the physicians than the physiotherapists (84.3% vs 61.7%, *P* < 0.001). The physiotherapists recorded better scores than the physicians on the physical activity promotion feasibility items that focused on *‘Separate one-on-one consultation’* (70.2% vs 54.9%, *P* = 0.017) and *‘Group session’* (75.5% vs 57.2%, *P* = 0.004) as the feasible physical activity promotion strategies for their patients (Table 3).

### Differences in overall disposition to physical activity promotion

The mean composite scores for the physicians and physiotherapists were 78.5 ± 6.7 and 72.5 ± 7.9, respectively out of a possible score of 100. The composite score for the physicians was significantly better (*p* < 0.05) than for the physiotherapists (Table 2). No significant difference in knowledge, confidence, role perception, barriers and feasibility of physical activity promotion by any of the demographic characteristics within the groups was found. However, significant difference in the composite score among physicians by gender (*P* < 0.05) was observed, with male physicians having better score in overall disposition to physical activity promotion than their female counterparts. No similar gender difference was observed among the physiotherapists (not shown in table).

### Optimal physical activity recommendation

Specifically when asked which physical activity prescription they would recommend, 56.4% (*n* = 53) physiotherapists chose 30 min of moderately intense physical activity 3-5 days per week, while only 11.7% (*n* = 11) chose 15 min of moderately intense physical activity 5-6 times a week. For physicians, 90 (58.8%) respondents chose 30 min of moderately intense physical activity 3-5 days per week, while only 9.8% (*n* = 15) chose 15 min of moderately physical activity 5-6 days per week. No significant difference (*P* = 0.679) was found between physiotherapists and physicians on optimal physical activity recommendations in terms of intensities, duration and sessions per week (Not shown in table).

## Discussion

Recommending physical activity participation is as important for overall prevention of non-communicable diseases just as recommending tobacco cessation and a no salt diet in treating hypertension [1, 2]. Generally, physiotherapists and physicians are involved in the primary prevention of non-communicable diseases and are also involved in risk reduction for these diseases. These professionals are also more likely than other health personnel to be approached for advice about physical fitness and physical activity by patients or clients [19–22]. Most physiotherapists and physicians in the present study had fairly good knowledge of physical activity promotional messages, perceived physical activity promotion as their role, reported little barrier to physical activity promotion on their job and believed promoting physical activity is feasible in their practice.

The findings on this group of physicians and physiotherapists in northern Nigeria is similar to that of Shirley et al. [21], who observed that Australian physiotherapists had very good knowledge, experienced little barriers and believed promoting physical activity was feasible to them in their practice. It is also consistent with the findings of van der Ploeg et al. [20] and that of Lawlor et al. [19] which showed that Australian and United Kingdom physicians, respectively had very good knowledge, experienced little barriers and believed promoting physical activity was feasible to their work. Also, consistent with findings among their colleagues in Canada [27, 33, 34], the Nigerian physicians and physiotherapists in our study were confident in giving physical activity counseling.

While the scores on knowledge, confidence, role perception, barriers to physical activity promotion of physicians and physiotherapists were comparable, better disposition by the physicians than the physiotherapists in the present study was surprising because physiotherapists can be expected to show better expertise and disposition to physical activity more than the physicians by virtue of their training focus [30, 31]. It could be that that Nigerian physiotherapists in the present study were more focused on the traditional clinical duty for which they are seeing patients (e.g., low back pain, knee strain, stroke rehabilitation, etc.), while the Nigerian physicians tend to take a broader view of patients and therefore, more likely to think about the overall health benefits of physical activity than the physiotherapists. Perhaps, to be consistent with the 21^st^ century focus of physiotherapy practice [30, 35, 36], the curriculum of physiotherapy training programmes in Nigeria should be updated to reflect contemporary knowledge and evidence on the strategic importance of physical activity and health promotion as effective non-invasive physiotherapy interventions for combatting the pandemic of lifestyle conditions. However, there could be other potential reasons for this surprising finding. It could be that the physicians in our study being older have more years of professional and practice experience that made them more disposed to promoting physical activity in their practice than the relatively younger physiotherapists. Since no previous study has compared both group of health professionals on physical activity disposition, it could be important to explore such comparison in future studies on this topic. However, the significantly better disposition observed for the physicians group compared to the physiotherapists should be interpreted with caution. This is because the subscales used to generate the composite disposition score may reflect different dimensions, and may limit the construct validity of the disposition scale. Also, the minimally important difference for the instrument is unknown and the higher disposition by physicians represented by only a 3-point difference, may not actually constitute a meaningful clinical difference.

In addition to our finding that some of the health professionals (between 21% to 31%) have poor knowledge of physical activity messages, just over half of the physicians (58.8%) and physiotherapists (56.4%) chose 30 min of moderate-intensity physical activity 3-5 times a week as the optimal physical activity to be recommended to their patients. This suggests that many of the physiotherapists and physicians do not have optimal knowledge on the intensity, frequency and duration of physical activity that could confer health benefits on their patients. International guidelines suggest that for health benefits, at least 150 min of moderate-intensity or 75 min of vigorous-intensity physical activity should be accumulated per week [13], and this can be accumulated in batches of 30 min per day of moderate intensity activity for 5 days in a week [11] or 20 min per day of vigorous intensity activity for 3 days in a week [12]. Our finding that showed male physicians have better disposition to physical activity promotion than their female counterparts was not in exact agreement with the finding in the study of Petrella et al. [33], which showed that female physicians make recommendations on physical activity to their patients more frequently than their male counterparts. However, absence of a significant difference between physical activity promotion dispositions among physiotherapists by gender as observed in the present study is consistent with that reported in another study of Australian physiotherapists [21].

Overall, identification of items differences between the physiotherapists and physicians is an indication that profession specific interventions should be prioritized when designing programs to improve physical activity disposition among physicians and physiotherapists in Nigeria. Perhaps, improving the knowledge of intensity of physical activity for health benefits is an important deficit that should be improved among Nigerian physiotherapists for effective physical activity promotion among their patients. It seems, education and training interventions that focus on improving confidence in suggesting specific physical activity programs for patients, enhancing feasibility of separate one-on one consultation and group session for physical activity promotion, and eliminating the barrier of lack of time could be most relevant areas for improvement among Nigerian physicians.

### Limitations and strength of the study

Social desirability phenomenon [37] in which the participants may have responded to the items on physical activity promotion in ways that is perceived to be professionally desirable could limit the results of the present study. It is possible that the physicians and physiotherapist may have exaggerated their perceived role, confidence, and feasibility of promoting a physically active lifestyle among their patients. In addition, the cross-sectional study design, relatively small sample size and sampling of convenience technique utilized could limit generalization of findings to other Nigerian physicians and physiotherapists of different characteristics from the present sample. While the study findings may be generalized to government hospitals, such generalization may not be applicable to physicians and physiotherapists practicing in non-government health institutions and private clinics or hospitals in Nigeria. Also, the validity of the modified questionnaire among Nigerian physicians and physiotherapists is unknown. The general absence of a significant difference in the mean summary scores for subscale variables between the two professions should also be interpreted with caution as this could reflect a ceiling effect resulting from the high scores reported by both physicians and physiotherapists. A strength of this study is that it was the first to compare the disposition of physiotherapists and physicians to physical activity promotion. It identified important profession-specific deficits that could be targeted when designing effective training programs to improve knowledge, role perception and feasibility, and barriers to physical activity promotion among Nigerian physiotherapists and physicians.

## Conclusions

This study shows that physiotherapists and physicians in Nigeria had fairly good knowledge of physical activity promotion, perceived few barriers to physical activity promotion, and reported physical activity promotion as part of their professional role and practice area. They were also confident in their ability to discuss and recommend physical activity promotion and believed promoting physical activity was feasible for them. A greater proportion of physiotherapists than physicians believed it is part of their role to suggest to patients to increase their daily physical activity and were also more confident in suggesting specific physical activity programs for their patients, but the physicians had better overall disposition to promoting physical activity than the physiotherapists. Overall, this study shows that although physiotherapists and physicians are positively disposed to physical activity promotion among their patients, some knowledge gaps exist on the recommendations for optimum physical activity for health enhancing benefits to patients. Nonetheless, these findings suggest that physiotherapists and physicians can serve as good advocates for optimizing outcome of physical activity promotion in Nigeria. However, for effective health promotion, prevention of non-communicable diseases and primary health care in Nigeria, these health professionals may require some training to enhance their knowledge, confidence and role feasibility in promoting health enhancing physical activity.

## Additional file

Additional file 1: Physical Activity Promotion Questionnaire. (DOC 64 kb)
