# Supplementary material for: A survey of physicians and physiotherapists on physical activity promotion in Nigeria
Source: Arch Physiother. 2017 May 19;7:5. doi: 10.1186/s40945-017-0034-8 (PMC5759899; doi:10.1186/s40945-017-0034-8)
Supplement: Additional file 1: — Physical Activity Promotion Questionnaire. (DOC 64 kb) [file 40945_2017_34_MOESM1_ESM.doc]

**Additional file 1**

**SECTION A**: **BIODATA AND PHYSICAL ACTIVITY RECOMMENDATIONS**

**BIODATA**

1. Age: _____________________
2. Gender: __________________
3. Years of work experience: _________________________________________________________
4. Average number of patients you see each week: _______________________________________
5. Usual number of hours worked each week: ___________________________________________
6. Your specialization:_______________________________________________________________
7. Your professional rank_____________________________________________________________
8. The Hospital where you work:________________________________________________________

**PHYSICAL ATIVITY RECOMMENDATIONS**

1. Are you aware of any Physical Activity Recommendation for Nigerian adults? ⃝ Yes ⃝ No
2. Which of the following best describe the optimal Physical activity that you will recommend for adults?

⃝ 10 minute each of moderately intense physical activity 5-6 days per week

⃝ 20 minute each of moderately intense physical activity 3-5 days per week

⃝ 30 minute each of moderately intense physical activity 3-5 days per week

⃝ 15 minute each of moderately intense physical activity 5-6 days per week

**SECTION B: PHYSICAL ACTIVITY PROMOTION**

Physical Activity: includes any activity from a low intensity level, such as walking, to a high intensity level, such as playing a competitive sport.

1. How often did you encourage your patients to have a more physically active lifestyle (beyond therapeutic exercise) in the last month? (please tick one)

- Never
- Rarely, only 1 or 2 patients
- Sometimes, perhaps 3-5 patients
- Often, perhaps 6-9 patients
- More often, 10 or more patients

1. To what extent do you agree or disagree with the following statements: (*circle only one for each statement*)

|  | **Strongly**  **disagree** | **disagree** | **Not**  **Sure** | **Agree** | **Strongly**  **Agree** |
| --- | --- | --- | --- | --- | --- |
| 1. Taking the stair at work and generally being more active each day is enough physical activity to improve health | 1 | 2 | 3 | 4 | 5 |
| 1. Half an hour of working on most days is all the physical activity that is needed for good health | 1 | 2 | 3 | 4 | 5 |
| 1. Physical activity that is good for health must make you puff and pant | 1 | 2 | 3 | 4 | 5 |
| 1. Several short walks of 10 minutes each on most days is better than one session of golf or soccer per week for good health | 1 | 2 | 3 | 4 | 5 |
| 1. Discussing the benefits of a physically active lifestyle with patients is part of my role | 1 | 2 | 3 | 4 | 5 |
| 1. Suggesting to patients ways to increase daily physical activity is part of my role | 1 | 2 | 3 | 4 | 5 |
| 1. I should be physically active to act as a role model for my patients | 1 | 2 | 3 | 4 | 5 |
| 1. I feel confident in in giving general advice to patients on physically active lifestyle | 1 | 2 | 3 | 4 | 5 |
| 1. If feel confident in suggesting specific physical activity programs for my patients | 1 | 2 | 3 | 4 | 5 |

1. How often does the following prevent you from promoting a physically active lifestyle in your patient (beyond therapeutic exercise)? (*circle only one for each statement*)

|  | **Never** | **Rarely** | **Sometimes** | **Often** | **Very often** |
| --- | --- | --- | --- | --- | --- |
| 1. Lack of time | 1 | 2 | 3 | 4 | 5 |
| 1. Lack of counselling skills | 1 | 2 | 3 | 4 | 5 |
| 1. Lack of remuneration for promoting physical activity | 1 | 2 | 3 | 4 | 5 |
| 1. Lack of interest in promoting physical activity | 1 | 2 | 3 | 4 | 5 |
| 1. Feeling it would not change the patient’s behavior | 1 | 2 | 3 | 4 | 5 |
| 1. Feeling it would not be beneficial for the patient | 1 | 2 | 3 | 4 | 5 |
| 1. Other:______________________________ | 1 | 2 | 3 | 4 | 5 |

1. What kind of physical activity promotion (beyond therapeutic exercise) would be feasible for you to your patients?(*circle only one for each statement*)

|  | **Highly**  **Feasible** | **Somewhat**  **Feasible** | **Not**  **Sure** | **Not**  **Really**  **Feasible** | **Totally**  **Unfeasible** |
| --- | --- | --- | --- | --- | --- |
| Brief counselling integrated into your regular consultation | 5 | 4 | 3 | 2 | 1 |
| Separate one-on-one consultations | 5 | 4 | 3 | 2 | 1 |
| Group session | 5 | 4 | 3 | 2 | 1 |
| Distribution of resources (e.g., brochures) | 5 | 4 | 3 | 2 | 1 |
